# Spiderweb-Like Fe-Co Prussian Blue Analogue Nanofibers as Efficient Catalyst for Bisphenol-A Degradation by Activating Peroxymonosulfate

**DOI:** 10.3390/nano9030402

**Published:** 2019-03-10

**Authors:** Hongyu Wang, Chaohai Wang, Junwen Qi, Yubo Yan, Ming Zhang, Xin Yan, Xiuyun Sun, Lianjun Wang, Jiansheng Li

**Affiliations:** 1 Key Laboratory of New Membrane Materials, Ministry of Industry and Information Technology, Nanjing University of Science & Technology, Nanjing 210094, China; wanghy4113@163.com (H.W.); wch2016@njust.edu.cn (C.W.); mzhang925@njust.edu.cn (M.Z.); 18205150297@163.com (X.Y.); sunxyun@njust.edu.cn (X.S.); wanglj@njust.edu.cn (L.W.); 2 Jiangsu Key Laboratory of Chemical Pollution Control and Resources Reuse, School of Environmental and Biological Engineering, Nanjing University of Science & Technology, Nanjing 210094, China; 3 Jiangsu Engineering Laboratory for Environment Functional Materials, Huaiyin Normal University, Huaian 223300, China; yubo.yan@outlook.com

**Keywords:** Fe-Co Prussian blue analogues, electrospinning, peroxymonosulfate, BPA

## Abstract

Prussian blue and its analogues (PBA) based nanomaterials have been widely applied to removing pollutants in the recent years. However, easy aggregation and poor recycling largely limit their practical applications. In this work, spiderweb-like Fe-Co Prussian blue analogue/polyacrylonitrile (FCPBA/PAN) nanofibers were prepared by electrospinning and applied to degrading bisphenol-A (BPA) by activating peroxymonosulfate (PMS). Detailed characterization demonstrated that a high loading of FCPBA (86% of FCPBA in FCPBA/PAN) was successfully fixed on the PAN nanofibers. 67% of BPA was removed within 240 min when 500 mg·L^−1^ PMS and 233 mg·L^−1^ FCPBA/PAN were introduced in 20 mg·L^−1^ BPA solution at initial pH of 2.8. Electron paramagnetic resonance (EPR) and radical inhibition experiments were performed to identify the possible degradation mechanism. For comparison, a low loading of FCPBA nanofibers (0.6FCPBA/PAN nanofibers, 43% of FCPBA in FCPBA/PAN) were also prepared and tested the catalytic performance. The results showed that the activity of FCPBA/PAN was much higher than 0.6FCPBA/PAN. Furthermore, a FCPBA/PAN packed column was made as a reactor to demonstrate the reusability and stability of FCPBA/PAN nanofibers, which also exhibited the bright future for the industrial application. This work makes it possible to fabricate efficient PBA nanocatalysts with excellent recyclability and promotes the application of PBA in industrial areas.

## 1. Introduction

Environmental pollution has received more and more attention nowadays due to the fast growth of economy and industry [1,2,3]. Pollutants, especially, the organic pollutants in water bodies have caused severe environmental and health issues because of the remarkable toxicity and persistent features [4,5,6,7]. So far, many approaches such as adsorption [8,9], biological treatment [10,11], and advanced oxidation processes (AOPs) [12,13,14,15,16] have been proposed for the removal of organic contaminants. Among them, SO_4_^•^^−^ based AOPs have undergone extensive study due to the high oxidation potential (2.5–3.1 V), wide suitable pH range (2–9), great resistance toward acid and base, and relative long life time (30–40 μs) [3,17,18]. Up until now, various transition metal ions and transition metal oxides were exploited for PMS activation [19,20,21,22]. However, these catalysts usually face the problem of high cobalt leaching and bring secondary pollution.

Recently, metal organic frameworks (MOFs) and porous coordination polymers (PCPs) used for peroxymonosulfate (PMS) activation have attracted great interest for their fascinating properties, for instance high surface area, high porosity, and adjustable pore structure [23,24,25]. Prussian blue and its analogues (PBAs) are a typical kind of PCPs and they have undergone extensive study recently. For instance, Lin’s group synthesized a series of Prussian blue analogues, and used them to activate peroxymonosulfate (PMS), which shows very high catalytic activity [26]. Zeng et al. reported the core-shell Fe-Co-Co Prussian blue analogues@poly(m-phenylenediamine) nanoparticles and used it to degrade Rhodamine B (RhB) and acquired an efficient performance [27]. However, the inherent natures of nanoparticle-based catalysts (easy to aggregate, hard to separate and potential environmental toxicity) constrain the practical engineering application. In order to solve these challenges, immobilized the nanoparticles into the certain shape (beads, tubes, or films) is a feasible way [28,29,30]. However, the trade-off effect between the amount of active sites and the shape of catalyst is difficult to balance.

So it is meaningful to find a facile and efficient method for the immobilization of nanocatalysts. Electrospinning is a facile way to immobilize MOFs on nanofibers and have been published in many literatures [31,32,33,34,35]. The high surface area of nanofibers ensures a high loading of nanoparticles. Recently, Wang et al. prepared MOFilters via electrospinning, which exhibited high removal efficiencies for PM2.5 and PM10 [36]. More recently, our group synthesized ZIF-67/PAN nanofibers for PMS activation, and a very high catalytic activity was acquired [37]. Kim et al. prepared Prussian blue/PAN (PB/PAN) nanofibers and used it to remove cesium [38]. However, in these studies, the loading amount of nanoparticles was relatively low (usually under 50%). Most of the nanoparticles were packed in the nanofibers, active sites could not be abundantly exposed to the outside, which to some extent restricted their performance. Herein, we firstly synthesized the spiderweb-like Fe-Co Prussian blue analogues/PAN nanofibers (FCPBA/PAN) with a high FCPBA loading (86% of FCPBA in FCPBA/PAN) via tuning the ratio of FCPBA and PAN. The catalytic performance of FCPBA/PAN was evaluated by degrade BPA in aqueous solution. For comparison, two other catalysts, a low loading of FCPBA nanofibers (0.6FCPBA/PAN nanofibers, 43% of FCPBA in FCPBA/PAN) and FCPBA nanoparticles, were also tested. To illustrate the synergistic effect of Co and Fe, single metal (Fe-Fe and Co-Co) Prussian blue analogues were synthesized and fabricated into nanofibers by electrospinning method. Furthermore, factors influencing the degradation efficiency were also considered, such as the loading of FCPBA/PAN, the dosage amount of PMS, BPA concentration, the effect of pH value and temperature, and competitive ions. Moreover, the degradation mechanism and synergistic effect of Fe and Co were studied. In addition, a FCPBA/PAN packed column was made as a reactor to demonstrate the reusability and stability of FCPBA/PAN nanofibers.

## 2. Experimental

### 2.1. Chemicals

Polyvinyl pyrrolidone (PVP, K30), Cobalt acetate (Co(CH_3_COO)_2_·4H_2_O), potassium hexacyanoferrate trihydrate (K_4_[Fe(CN)_6_]·3H_2_O) were all purchased from Sinopharm Chemical Reagent Co., Ltd. (Shanghai, China) Polyacrylonitrile (PAN), potassium peroxymonosulfate (PMS), methanol and anhydrous ethanol were purchased from Sigma-Aldrich (Shanghai, China). Ferrous sulfate heptahydrate (FeSO_4_·7H_2_O), tertbutyl alcohol (TBA) and N,N-dimethylformamide (DMF) were acquired from Nanjing Chemical Reagent Co., Ltd. (Nanjing, China) Potassium hexacyanocobaltate(III) (K_3_[Co(CN)_6_]) was purchased from Alfa Aesar (Ward Hill, MA, USA). Bisphenol A (BPA) was purchased from Aladdin (Shanghai, China). Hydrochloric acid was purchased from Shanghai Lingfeng Chemical Reagent Co., Ltd. (Shanghai, China).

### 2.2. Synthesis of (Prussian Blue Analogue) PBA Nanoparticles

FCPBA, Co-Co PBA nanoparticles were synthesized via a previous published method [39]. Typically, 0.332 g K_4_[Fe(CN)_6_]·3H_2_O was dissolved in 200 mL of DI water with continuous stirring for 10 min, 6 g PVP was added into it and stirred for another 20 min. Then a peristaltic pump was used to drop 200 mL of 2.1 mg·mL^−1^ FeSO_4_·7H_2_O or 1.87 mg·mL^−1^ Co(CH_3_COO)_2_·4H_2_O solution in the above solution with a flow rate of 4 mL·min^−1^. FCPBA and Co-Co PBA were acquired after stirring 24 h under room temperature. To get Fe-Fe PBA, 15.2 g PVP was put into 200 mL of DI water with continuous stirring. When PVP was completely dissolved, 1.8 mL of hydrochloric acid was dropped and the mixture was stirred for 10 min. Then 0.44 g K_4_[Fe(CN)_6_]·3H_2_O was added into the mixture and stirred for another 1 h. The resultant was then transferred into a polytetrafluoroethylene (PTFE) lined reactor, heated under 80 °C for 24 h. After centrifugation, the precipitate was washed with DI water and anhydrous ethanol for 3 times for further purification and put in vacuum drying oven for 6 h at 60 °C.

### 2.3. Fabrication of Fe-Co Prussian Blue Analogue/Polyacrylonitrile (FCPBA/PAN) Nanofiber

In a typical electrospinning experiment, 0.36 g FCPBA was well dispersed in 2 mL DMF solution with vigorous sonication. After that, 0.06 g PAN was put into the above solution and stirred for 3 h at 60 °C to acquire the precursor for electrospinning. The well dispersed suspension was through metallic needle by an injection pump at a fixed speed (0.1 mm·min^−1^) and applied with a positive voltage of 8 kV. 

### 2.4. Characterization

The morphology of nanoparticles and nanofibers were conducted on a Scanning electron microscopy (SEM) (FEI Quanta 250F, FEI, Hillsboro, OR, USA) at 20 kV. Transmission electron microscopy (TEM) was acquired from a FEI Tecnai 20 electron microscope (FEI, Hillsboro, OR, USA) at 200 kV. X-ray diffraction (XRD) were analyzed on a BRUKER D8 (BRUKER, Shanghai, China), Cu Kα at 40 kV and 40 mA (λ = 1.5418 Å). Thermogravimetric analysis (TGA) was carried out on a SDTQ 600 analyzer (TA Instruments, New Castle, DE, USA) in nitrogen flow. Infrared spectra (IR) was determined on a Perkin Elmer 100 (PERKINELMER, Waltham, MA, USA).

### 2.5. Catalytic Activity Measurements

In this study, BPA was used as a typical pollutant to evaluate the performance of FCPBA/PAN. Typically, 100 mL of BPA solution (20 mg·L^−1^) was prepared. PMS (50 mg) was added into it and stirred to make sure it was completely dissolved. Then 23.3 mg of FCPBA/PAN was added into the reaction system. 1.5 mL of solution was carried out and quenched with 1.5 mL methanol at selected time intervals. The samples were tested by high-performance liquid chromatograph (HPLC) (Waters e2695, Waters, Milford, MA, USA). TOC tester (Vario TOC, Elementar, Elementar, Shanghai, China) was used to test the total organic carbon (TOC) amount of the samples. The electron paramagnetic resonance (EPR) examination was carried on a Bruker EMX 10/12 spectrometer (BRUKER, Shanghai, China) to determine the signals of radicals.

## 3. Results and Discussion

### 3.1. Synthesis and Characterization of FCPBA/PAN

**Scheme 1:**
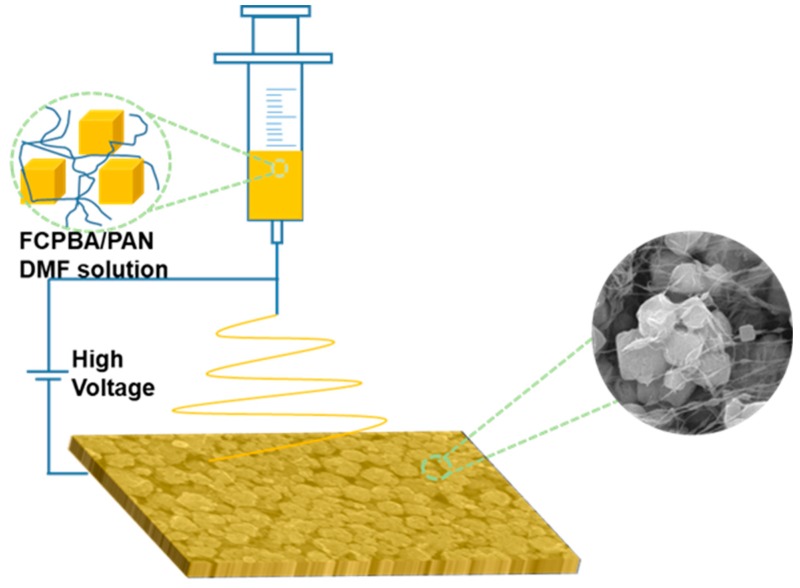
Illustration of the preparation process of the Fe-Co Prussian blue analogue/polyacrylonitrile (FCPBA/PAN) nanofibers.

**Figure 1:**
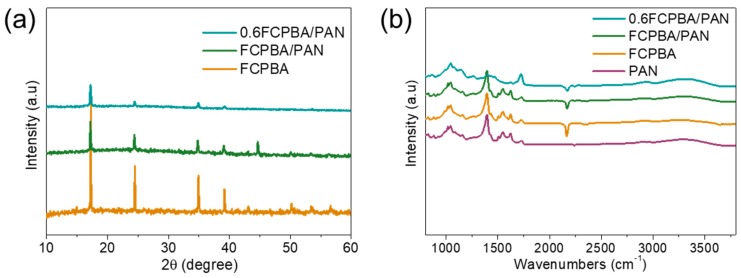
(**a**) X-ray diffraction (XRD) patterns, (**b**) Infrared Spectroscopy (IR) spectra of FCPBA, PAN, FCPBA/PAN, and 0.6FCPBA/PAN.

Scheme 1 shows the fabrication of FCPBA/PAN nanofiber. Firstly, the beforehand synthesized FCPBA was well dispersed in DMF solution with vigorous sonication. Then PAN was added into it and stirred for 3 h at 60 °C to form a uniform FCPBA/PAN/DMF homogeneous solution. Then the prepared precursor was transformed into FCPBA/PAN nanofiber via electrospinning. XRD peaks showed that the prepared FCPBA was in accordance with the simulated FCPBA, suggesting the high purity of the synthesized FCPBA (Figure 1a). Furthermore, the characteristic diffraction peaks of FCPBA/PAN and 0.6FCPBA/PAN were consistent with FCPBA, indicating that the synthesized FCPBA was fixed on PAN nanofibers. The IR spectra (Figure 1b) also proved the immobilization of FCPBA to PAN nanofibers. TG test was carried to study the thermal stability of the FCPBA/PAN nanofibers (Figure S1). The samples were heated under N_2_ atmosphere, and about 18% weight loss from 20 to 130 °C of FCPBA/PAN was due to the dehydration of water molecules. The morphology of FCPBA and FCPBA/PAN were further verified by SEM and TEM.

**Figure 2:**
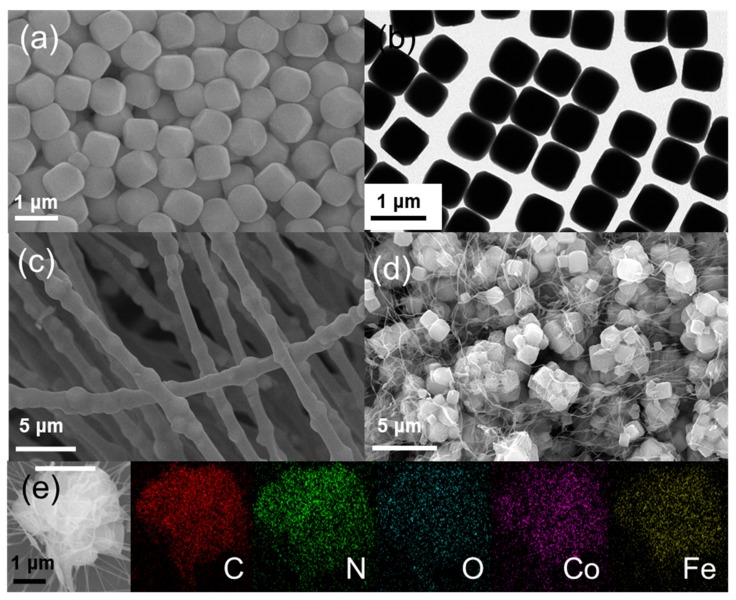
(**a**) SEM image and (**b**) TEM image of FCPBA; (**c**,**d**) SEM images of 0.6FCPBA/PAN and FCPBA/PAN; (**e**) Energy Dispersive Spectrometer (EDS) elemental mapping of FCPBA/PAN.

Figure 2a shows the SEM image of FCPBA, from the picture we can see that the morphology of the as-prepared FCPBA was very uniform as every nanoparticle has a cubic structure. TEM results (Figure 2b) further revealed morphology of the synthesized FCPBA, and the average particle size of FCPBA was approximately 800−900 nm. Furthermore, the particle size distribution was also conducted and results were shown in Figure S2a. The average particle size of FCPBA was about 813 nm. Besides, we tested the zeta potential and stability of FCPBA nanoparticles. Results showed the zeta potential changing of FCPBA nanoparticles when pH value increased from 3 to 10. The isoelectric point of FCPBA was acquired at around 8.4 (Figure S2b). Figure 2c shows the SEM image of 0.6FCPBA/PAN. From the picture we can see that a low loading of FCPBA was fixed into the nanofibers. Figure 2d shows the SEM image of FCPBA/PAN. This showed that the FCPBA/PAN nanofibers were composed of numerous microspheres. With the concentration of FCPBA increased, PAN cannot pack the nanoparticles completely. A few particles agglomerated together and were outside among the nanofibers, forming a microsphere and microspheres binding each other with PAN nanofibers. The Energy Dispersive Spectrometer (EDS) elemental mapping further confirmed the distribution of FCPBA nanoparticles in the microsphere (Figure 2e). 

Figure S3a,b were the nitrogen adsorption/desorption isotherm and pore composition of FCPBA/PAN. From Figure S3a, we can see that the sample showed type IV isotherms and when relative pressure was 0.1, a discontinuous hysteresis loop occurred. The appearance was caused by the sorption characteristic of polymer [40]. Figure S3b revealed the distribution of micropores and mesopores in FCPBA/PAN. Besides, Brunauer–Emmett–Teller (BET) surface areas reached 163.3 m^2^/g, which was conducive to acquiring a high catalytic activity. The hydrophilic property was examined and Figure S4 was the result of water contact angle. The picture showed that FCPBA/PAN nanofibers were hydrophilic and the water contact angle was 44°. As shown in Figure S5, the FCPBA/PAN nanofibers were very flexible and can be easily made into devices.

### 3.2. Activation of PMS by FCPBA/PAN

**Figure 3:**
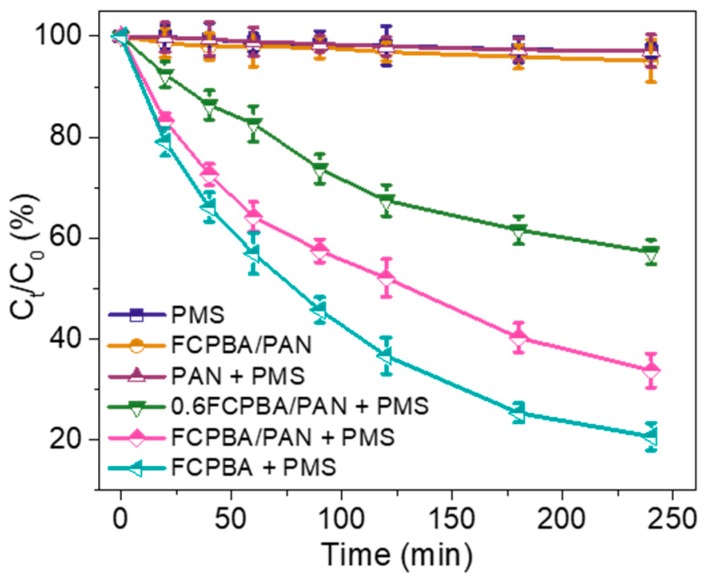
Bisphenol-A (BPA) removal in different reaction systems, (BPA solution = 20 mg·L^−1^; peroxymonosulfate (PMS) = 500 mg·L^−1^; FCPBA/PAN = 233 mg·L^−1^, containing 200 mg·L^−1^ of FCPBA; FCPBA = 200 mg·L^−1^; PAN = 200 mg·L^−1^; 0.6FCPBA/PAN = 466 mg·L^−1^; containing 200 mg·L^−1^ of FCPBA; initial pH = 2.8; T = 293 K).

**Figure 4:**
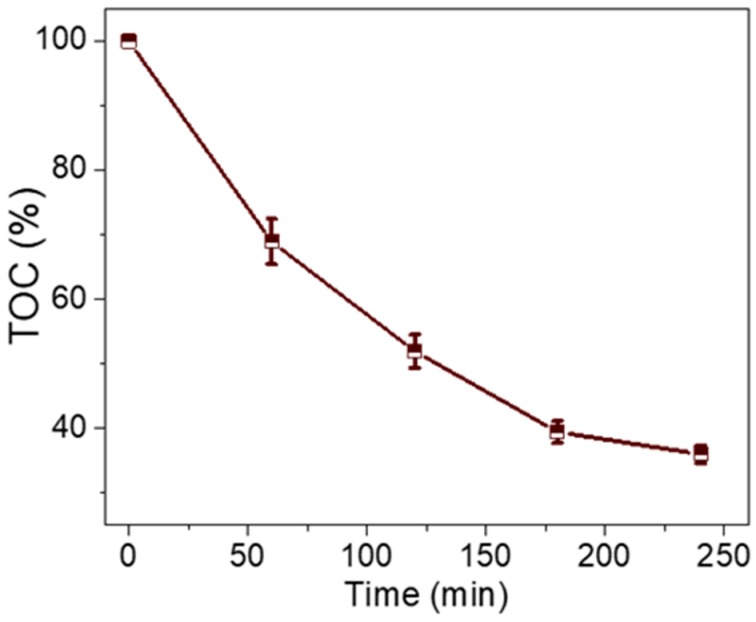
Total organic carbon (TOC) removal during the degradation of BPA activated by FCPBA/PAN (BPA solution = 20 mg·L^−1^; PMS = 500 mg·L^−1^; FCPBA/PAN = 233 mg·L^−1^; initial solution pH = 2.8; T = 293 K).

Degradation of BPA was chosen to investigate the performance of catalysts and the results were shown in Figure 3. When FCPBA/PAN was added to BPA solution without the addition of PMS, less than 3% of BPA was removed by static adsorption after 240 min, which demonstrated that the adsorption of FCPBA/PAN was very weak. Meanwhile, using only PMS showed a degradation of less than 5%, suggesting that active radicals could hardly be generated by PMS alone. When the pure PAN nanofiber and 0.6FC/PAN were added into the BPA solution which contained 500 mg/L of PMS, only 5% and 40% of BPA was degraded after 240 min. As a comparison, FCPBA/PAN and FCPBA nanoparticles were conducted at the same condition and reached a higher removal efficiency of 67% and 79%, respectively, indicating that more active sites exposed to reaction system could contribute to the catalytic performance. Total organic carbon (TOC) of BPA was also tested and result was shown in Figure 4. About 65% of BPA was degraded to CO_2_ and H_2_O after 240 min, this result was caused by some degradation intermediates that were hard to be degraded, which was close to the previous report [37,41]. The particle stability was tested by dropping FCPBA into reaction solution for 4 h. XRD result showed that the structure of FCPBA still maintained well after catalyzation (Figure S2c).

### 3.3. Effect of pH on the Degradation of BPA

**Figure 5:**
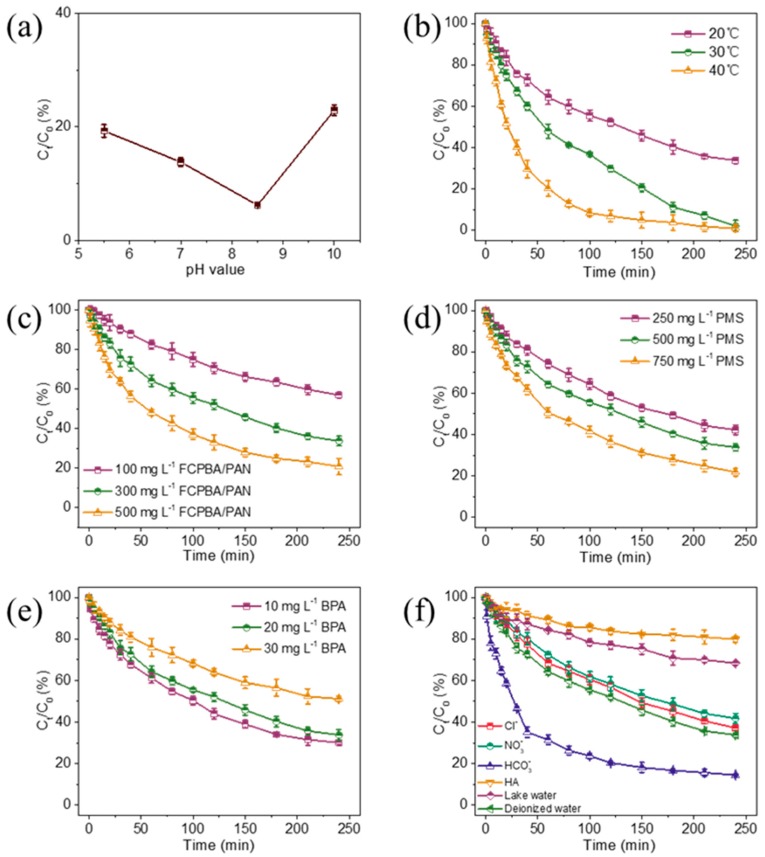
Effects of (**a**) different initial pH, (**b**) reaction system temperatures, (**c**) catalyst dosages, (**d**) PMS dosages, (**e**) BPA concentrations, and (**f**) ions and organics on the BPA removal efficiency. Reaction conditions: BPA solution = 20 mg·L^−1^ (for a, b, c, d, and f); PMS = 500 mg·L^−1^ (for a, b, c, e, and f); FCPBA/PAN = 233 mg·L^−1^ (for a, b, d, e, and f); T = 293 K (for a, c, d, e, and f); initial solution pH = 2.8 (for b, c, d, e, and f).

The effect of pH displayed a noteworthy role in the reaction system, so it is indispensable to study the influence of initial pH in the process of PMS oxidation. Therefore, different pH values ranging from 5.5 to 10 were investigated in the degradation system (Figure 5a). The removal efficiency has a notable enhancement with the initial pH increased from 5.5 to 8.5, and the highest removal rate was acquired when the pH value was 8.5. About 94% of BPA was removed after 240 min. However, when initial pH still increased, the removal efficiency was decreased, this may be ascribed to the self-decomposition of PMS (HSO_5_^−^ react with OH^−^), and thus restricting the BPA degradation [40]. These results showed that weak basic condition is helpful to promote the catalytic efficiency, meanwhile acid and strong alkaline conditions could restrain the catalytic reaction process [42].

### 3.4. Effect of Temperature on the Degradation of BPA

Besides pH, the solution temperature is also critical for reaction system. Hence, a series of temperatures (20, 30, and 40 °C) was chosen for investigation. The efficiency of BPA removal was obviously enhanced (Figure 5b). More than 90% of BPA was degraded within 100 min at 40 °C. A first-order rate equation [43] was used to study the kinetics for the degradation of BPA (Equation (1)):

(1) $$\ln\frac{c_{t}}{c_{0}}=-kt$$

where k denotes the rate constant and $c_{0}$ and $c_{t}$ are the initial and real-time concentration of BPA solution. The rate constants were 0.0045, 0.013, and 0.019 min^−1^ corresponding to the reaction system temperatures of 20 to 40 °C. According to Arrhenius equation, the k values under different temperatures was applied for determining the activation energy (Equation (2)):

(2) $$\ln k=\ln A-\frac{E_{a}}{\mathrm{RT}}$$

where *E*_a_ denotes the Arrhenius activation energy (kJ·mol^−1^), A denotes the Arrhenius factor, R represents the gas constant (8.314 J·mol^−1^·K^−1^), and T is the solution temperature (K). The activation energy ($E_{a}$) is 49.88 kJ·mol^−1^ and the value is smaller than the published article, suggesting that the removal efficiency of BPA is very good [42].

### 3.5. Effects of Catalyst Dosage, PMS Dosage and BPA Concentration

The dosage of FCPBA/PAN was tested via controlling the amount of FCPBA/PAN in the BPA solution, and result was shown in Figure 5c. With the FCPBA/PAN dosage increased from 100 mg·L^−1^ to 500 mg·L^−1^, the catalytic performance has a conspicuous improvement. When the FCPBA/PAN dosage was 500 mg·L^−1^, about 80% of BPA was degraded within 240 min, which indicate that a higher catalyst loading benefits the PMS activation [44].

The effect of PMS dosage was also studied via changing the amounts of PMS in the BPA solution. As shown in Figure 5d, when PMS was only 250 mg·L^−1^, barely 58% of BPA in water was degraded and the removal rate reached 80% when PMS dosage was 250 mg·L^−1^. The result demonstrated that a sufficient amount of PMS was required during the degradation of BPA and also confirmed the high efficiency of FCPBA/PAN.

Furthermore, different concentration of BPA were also investigated (Figure 5e). Obviously, near 70% of BPA was degraded within 240 min in 10 mg·L^−1^ of BPA solution, suggesting that an enhanced degradation performance can be acquired in a low concentration. However, 50% of BPA was degraded in 30 mg·L^−1^ of BPA solution attributing to the influence of low PMS dosage that was lacking enough SO_4_^•^^−^.

### 3.6. Effect of Anions and Organics

Wastewater or natural water body usually contains salts and organics, so it’s necessary to investigate the effect of anions (Cl^−^, HCO_3_^−^, and NO_3_^−^) and organics [45]. Figure 5f shows BPA degradation under various conditions. The degradation efficiency has a slight decrease with the existence of Cl^−^ (10 mM), suggesting that the existence Cl^−^ has some influence on BPA degradation. When NO_3_^−^ (10 mM) was adding in the reaction system, the similar result can be found. Interestingly, when 10 mM HCO_3_^−^ was added in BPA solution, about 85% of BPA was degraded within 240 min. This phenomenon can be explained as the addition of HCO_3_^−^ changed the pH value of the solution. HA is chosen as a typical organics [46], and the removal rate of BPA is about 20% when existing 2 mg·L^−1^ of HA. The result may be explained by the generated radicals being consumed by HA. Furthermore, we also studied the removal efficiency under real water. The water sample was taken from Xuanwu Lake in Nanjing and the result shows that BPA in lake water was degraded slower than that in deionized water, which was owing to the consumption of SO_4_^•^^−^ by original organics in lake water.

### 3.7. Possible Degradation Mechanism

In the light of the related work [26,47,48], the possible degradation mechanism is as follows (Equations (3)–(6)):

Co^3+^ + HSO_5_^−^ → Co^2+^ + SO_5_^•^^−^ + H^+^ (3)

Co^2+^ + HSO_5_^−^ → Co^3+^ + SO_4_^•^^−^ + OH^−^ (4)

Fe^2+^ + HSO_5_^−^ → Fe^3+^ + SO_4_^•^^−^ + OH^−^ (5)

Fe^3+^ + HSO_5_^−^ → Fe^2+^ + SO_5_^•^^−^ + H^+^ (6)

Firstly, Co^2+^ and Fe^2+^ can be oxidized during the activation of PMS to generate SO_4_^•^^−^. Meanwhile, Co^3+^ and Fe^3+^ can react with SO_5_^•^^−^ to regenerate Co^2+^ and Fe^2+^. Additionally, SO_4_^•^^−^ derived from PMS can induce the formation of ^•^OH (Equation (7)):

SO_4_^•^^−^ + H_2_O → SO_4_^2^^−^ + OH^•^ + H^+^ (7)

**Figure 6:**
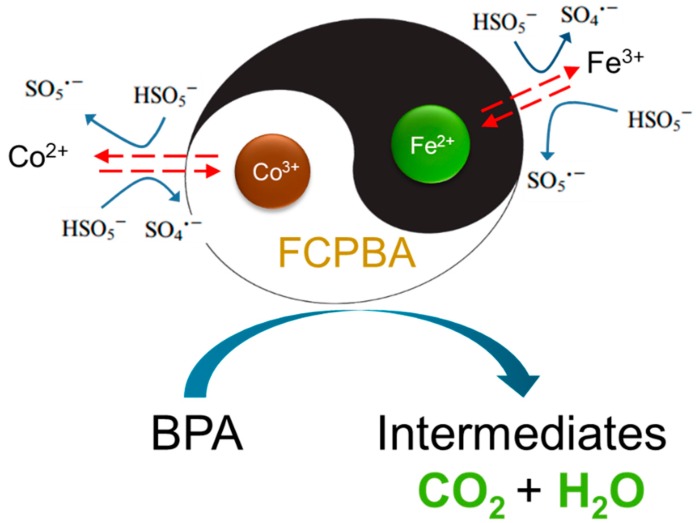
A proposed mechanism of the activation of PMS by FCPBA/PAN to generate sulfate radicals.

**Figure 7:**
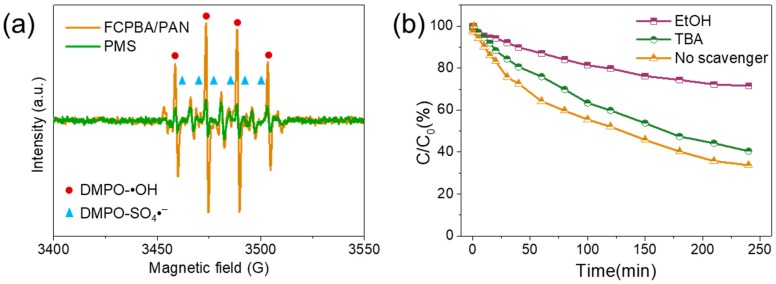
(**a**) Electron paramagnetic resonance (EPR) spectra of different systems in BPA solution; (**b**) effects of radical scavengers on the degradation of BPA. Reaction conditions: BPA solution = 20 mg·L^−1^, PMS = 500 mg·L^−1^, FCPBA/PAN = 233 mg·L^−1^, DMPO = 0.1 M, initial solution pH = 2.8, T = 298 K.

The generated SO_4_^•^^−^ has powerful oxidizing potential, which can oxidize BPA into the intermediates, CO_2_ and H_2_O (Figure 6). To further reveal the contributions of the generated radicals in the degradation of BPA, EPR experiments and radical inhibition experiment were performed (Figure 7a). Asthenic radical signal was collected when there was only PMS in the solution, which indicated that the absence of PMS alone can decompose itself slightly. However, when both FCPBA/PAN and PMS existed, a strong radical signal (intensity ratio 1:2:2:1) was acquired, which belonged to DMPO–^•^OH product (αH = αN = 14.9 G), indicating that ^•^OH was generated. The signals for DMPO–SO_4_^•^^−^ (αN = 13.2 G, αH = 9.6 G, αH = 1.48 G, and αH = 0.78 G) can also be found, which suggested that SO_4_^•^^−^ was generated.

Both the two radicals are efficient for the removal of BPA because of their high oxidizing potential [49,50]. To reveal the roles ^•^OH and SO_4_^•^^−^ plays in the degradation system, several kinds of quenching regents were dropped into BPA solution such like ethanol (mainly reacts with ^•^OH and SO_4_^•^^−^) and TBA (mainly reacts with ^•^OH) [51,52]. Results are shown in Figure 7b. When ethanol was used to quench ^•^OH and SO_4_^•^^−^, only 30% of BPA was degraded within 240 min. However, when TBA was added, the removal efficiency had a slightly decline. The EPR and radical inhibition together demonstrate both ^•^OH and SO_4_^•^^−^ have catalytic activity, and SO_4_^•^^−^ owns the main contribution to BPA degradation.

### 3.8. Synergistic Effect of Fe and Co

**Figure 8:**
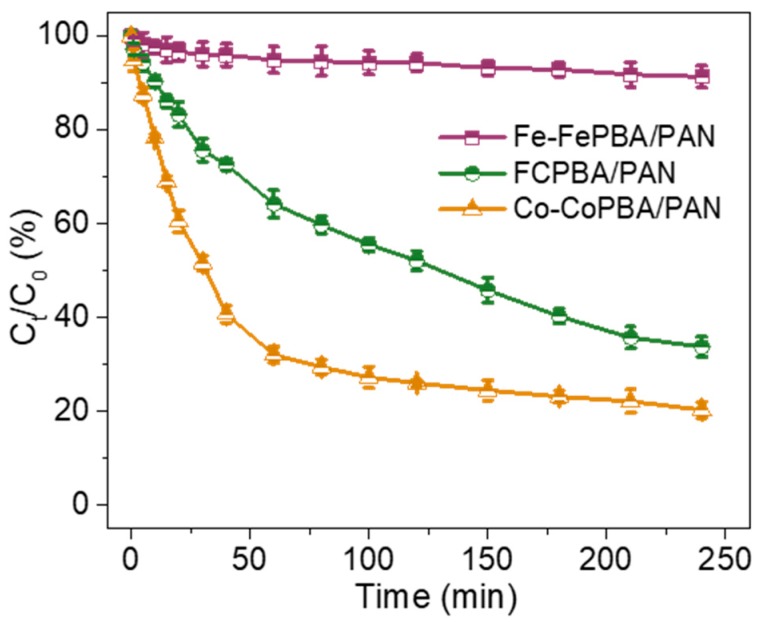
Synergistic effect of Fe and Co.

So as to demonstrate the synergistic effect of Fe and Co, Fe-Fe and Co-Co PBA were synthesized and fabricated into nanofibers. Figure S6 were the SEM and TEM images of Fe-Fe and Co-Co PBA. The comparative experiment was carried under the same condition and result was shown in Figure 8. When Fe-Fe PBA/PAN was put into BPA solution, little of BPA was degraded. Approximately 10% of BPA was removed within 240 min, indicating that Fe alone has poor catalytic activity towards PMS activation. Conversely, the removal efficiency attained 88% when Co-Co PBA/PAN was selected as the catalyst. When FCPBA/PAN was adopted in the degradation system, 65% of BPA was degraded after 240 min, demonstrating that Fe combined with Co can acquire a very good catalytic performance. Furthermore, cobalt leaching was determined by ICP-OES. As shown in Figure S7, the Co leaching of FCPBA/PAN was 0.17 mg·L^−1^ while it attained 0.67 mg·L^−1^ in Co-Co PBA/PAN after reaction, which demonstrate that Co combined with Fe can effectively suppress the Co leaching.

### 3.9. Reusability

**Figure 9:**
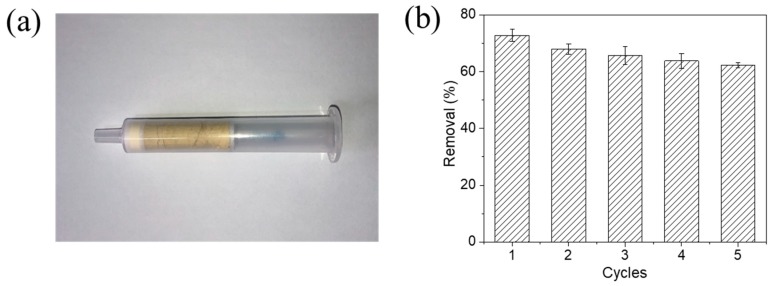
(**a**) Photos of FCPBA/PAN column; (**b**) cycle test of FCPBA/PAN in the activation of PMS to degrade BPA (BPA solution = 20 mg·L^−1^; PMS = 500 mg·L^−1^; FCPBA/PAN = 233 mg·L^−1^; pH = 3.2).

Reusability is also an important factor to evaluate catalyst and a recycling experiment was adopted to investigated the reusability of FCPBA/PAN [53,54]. To illustrate the reusability and the potential for practical application, a FCPBA/PAN packed column was designed (shown in Figure 9a). Both ends of the FCPBA/PAN column equipped with polyethylene sieve plates, which could prevent the catalyst wastage during the reaction process. Figure 9b demonstrated the reusability of FCPBA/PAN in five cycles. Firstly, the removal efficiency of FCPBA/PAN column could attain 72% when flow rate was 0.5 mL/min. And after five cycles, the removal efficiency still remained at 62%, which suggested that FCPBA/PAN nanofibers have good reusability and the designed FCPBA/PAN column has promising potential for practical application.

## 4. Conclusion

In conclusion, we reported a facile method to fabricate spiderweb-like Fe-Co Prussian blue analogue/PAN (FCPBA/PAN) nanofibers for PMS activation. The fabricated FCPBA/PAN nanofibers displayed good removal efficiency for BPA solution. Meanwhile, it can significantly suppress the cobalt leaching owing to the synergistic effect of Fe and Co and the high active sites. The as-prepared nanofibers have good reusability and stability. Furthermore, a FCPBA/PAN column was fabricated and showed satisfactory removal efficiency to target organic pollutant, which illustrated the potential for industrial applications. This work provides a simple and efficient way for MOF and PCP nanoparticles in environmental applications.

## Supplementary Materials

The following are available online at https://www.mdpi.com/2079-4991/9/3/402/s1, Figure S1: (Thermogravimetric analysis) TG curves of PAN, FCPBA, FCPBA/PAN, Figure S2: (a) Particle size; (b) zeta potential and (c) stability of FCPBA, Figure S3: (a) N2 sorption isotherms and (b) pore size distribution of FCPNA/PAN nanofibers, Figure S4: Water contact angle of FCPBA/PAN nanofibers, Figure S5: Mechanical properties of FCPBA/PAN nanofibers, Figure S6: SEM images of (a) Fe-Fe PBA and (b) Co-Co PBA; TEM imagers of (c) Fe-Fe PBA and (d) Co-Co PBA, Figure S7: Cobalt leaching of FCPBA/PAN and Fe-FePBA/PAN in reaction system.

## References

[1] Wacławek S., Lutze H.V., Grübel K., Padil V.V.T., Černík M., Dionysiou D.D. (2017). Chemistry of persulfates in water and wastewater treatment: A review. Chem. Eng. J..

[2] Liang P., Zhang C., Duan X., Sun H., Liu S., Tade M.O., Wang S. (2017). An insight into metal organic framework derived N-doped graphene for the oxidative degradation of persistent contaminants: Formation mechanism and generation of singlet oxygen from peroxymonosulfate. Environ. Sci. Nano.

[3] Oh W.D., Dong Z., Lim T.T. (2016). Generation of sulfate radical through heterogeneous catalysis for organic contaminants removal: Current development, challenges and prospects. Appl. Catal. B Environ..

[4] Ghanbari F., Moradi M. (2017). Application of peroxymonosulfate and its activation methods for degradation of environmental organic pollutants: Review. Chem. Eng. J..

[5] Hu P., Long M. (2016). Cobalt-catalyzed sulfate radical-based advanced oxidation: A review on heterogeneous catalysts and applications. Appl. Catal. B Environ..

[6] Wang J., Wang S. (2016). Removal of pharmaceuticals and personal care products (PPCPs) from wastewater: A review. J. Environ. Manag..

[7] Xiong Z., Lai B., Yang P. (2018). Insight into a highly efficient electrolysis-ozone process for N, N-dimethylacetamide degradation: Quantitative analysis of the role of catalytic ozonation, fenton-like and peroxone reactions. Water Res..

[8] Hasan Z., Jhung S.H. (2015). Removal of hazardous organics from water using metal-organic frameworks (MOFs): Plausible mechanisms for selective adsorptions. J. Hazard. Mater..

[9] Haque E., Jun J.W., Jhung S.H. (2011). Adsorptive removal of methyl orange and methylene blue from aqueous solution with a metal-organic framework material, iron terephthalate (MOF-235). J. Hazard. Mater..

[10] Bourgin M., Beck B., Boehler M., Borowska E., Fleiner J., Salhi E., Teichler R., Gunten U., Siegrist H., McArdell C.S. (2018). Evaluation of a full-scale wastewater treatment plant upgraded with ozonation and biological post-treatments: Abatement of micropollutants, formation of transformation products and oxidation by-products. Water Res..

[11] Grandclement C., Seyssiecq I., Piram A., Wong-Wah-Chung P., Vanot G., Tiliacos N., Roche N., Doumenq P. (2017). From the conventional biological wastewater treatment to hybrid processes, the evaluation of organic micropollutant removal: A review. Water Res..

[12] Li H., Tian J., Zhu Z., Cui F., Zhu Y.A., Duan X., Wang S. (2018). Magnetic nitrogen-doped nanocarbons for enhanced metal-free catalytic oxidation: Integrated experimental and theoretical investigations for mechanism and application. Chem. Eng. J..

[13] Jaafarzadeh N., Ghanbari F., Ahmadi M. (2017). Efficient degradation of 2,4-dichlorophenoxyacetic acid by peroxymonosulfate/magnetic copper ferrite nanoparticles/ozone: A novel combination of advanced oxidation processes. Chem. Eng. J..

[14] Mirzaei A., Chen Z., Haghighat F., Yerushalmi L. (2017). Removal of pharmaceuticals from water by homo/heterogonous Fenton-type processes—A review. Chemosphere.

[15] Tian W., Zhang H., Qian Z., Ouyang T., Sun H., Qin J., Tadé M.O., Wang S. (2018). Bread-making synthesis of hierarchically Co@C nanoarchitecture in heteroatom doped porous carbons for oxidative degradation of emerging contaminants. Appl. Catal. B Environ..

[16] Qi F., Chu W. (2015). Heterogeneous catalytic ozonation of phenacetin in water using magnetic spinel ferrite as catalyst: Comparison of surface property and efficiency. J. Mol. Catal. A-Chem..

[17] Qi F., Chu W., Xu B. (2014). Modeling the heterogeneous peroxymonosulfate/Co-MCM41 process for the degradation of caffeine and the study of influence of cobalt sources. Chem. Eng. J..

[18] Li J., Xu M., Yao G., Lai B. (2018). Enhancement of the degradation of atrazine through CoFe_2_O_4_ activated peroxymonosulfate (PMS) process: Kinetic, degradation intermediates, and toxicity evaluation. Chem. Eng. J..

[19] Anipsitakis G.P., Dionysiou D.D. (2003). Degradation of organic contaminants in water with sulfate radicals generated by the conjunction of peroxymonosulfate with cobalt. Environ. Sci. Technol..

[20] Du J., Bao J., Liu Y., Ling H., Zheng H., Kim S.H., Dionysiou D.D. (2016). Efficient activation of peroxymonosulfate by magnetic Mn-MGO for degradation of bisphenol A. J. Hazard. Mater..

[21] Zhang J., Chen M., Zhu L. (2016). Activation of persulfate by Co_3_O_4_ nanoparticles for orange G degradation. RSC Adv..

[22] Oh W.D., Dong Z., Hu Z.T., Lim T.T. (2015). A novel quasi-cubic CuFe_2_O_4_–Fe_2_O_3_ catalyst prepared at low temperature for enhanced oxidation of bisphenol A via peroxymonosulfate activation. J. Mater. Chem. A.

[23] Li H., Wan J., Ma Y., Wang Y., Chen X., Guan Z. (2016). Degradation of refractory dibutyl phthalate by peroxymonosulfate activated with novel catalysts cobalt metal-organic frameworks: Mechanism, performance, and stability. J. Hazard. Mater..

[24] Liu X., Li Y., Ban Y., Peng Y., Jin H., Bux H., Xu L., Caro J., Yang W. (2013). Improvement of hydrothermal stability of zeolitic imidazolate frameworks. Chem. Commun..

[25] Lin K.Y.A., Chang H.A. (2015). Zeolitic Imidazole Framework-67 (ZIF-67) as a heterogeneous catalyst to activate peroxymonosulfate for degradation of Rhodamine B in water. J. Taiwan Inst. Chem. Eng..

[26] Lin K.Y.A., Chen B.J., Chen C.K. (2016). Improvement of hydrothermal stability of zeolitic imidazolate frameworks. RSC Adv..

[27] Zeng L., Xiao L., Shi X., Wei M., Cao J., Long Y. (2019). Core-shell Prussian blue analogues@ poly(m-phenylenediamine) as efficient peroxymonosulfate activators for degradation of Rhodamine B with reduced metal leaching. J. Colloid. Interf. Sci..

[28] Li L., Yao J., Xiao P., Shang J., Feng Y., Webley P.A., Wang H. (2013). One-step fabrication of ZIF-8/polymer composite spheres by a phase inversion method for gas adsorption. Colloid Polym. Sci..

[29] Chen Y., Chen F., Zhang S., Cai Y., Cao S., Li S., Zhao W., Yuan S., Feng X., Cao A. (2017). Facile Fabrication of Multifunctional Metal-Organic Framework Hollow Tubes to Trap Pollutants. J. Am. Chem. Soc..

[30] Hou J., Sutrisna P.D., Zhang Y., Chen V. (2016). Continuous Metal-Organic Framework Membranes on Flexible Polymer Substrates. Angew. Chem. Int. Ed..

[31] Cai Y., Chen D., Li N., Xu Q., Li H., He J., Lu J. (2017). Nanofibrous metal–organic framework composite membrane for selective efficient oil/water emulsion separation. J. Membrane Sci..

[32] Armstrong M.R., Shan B., Maringanti S.V., Zheng W., Mu B. (2016). Hierarchical Pore Structures and High ZIF-8 Loading on Matrimid Electrospun Fibers by Additive Removal from a Blended Polymer Precursor. Ind. Eng. Chem. Res..

[33] Lu A.X., McEntee M., Browe M.A., Hall M.G., DeCoste J.B., Peterson G.W. (2017). MOFabric: Electrospun Nanofiber Mats from PVDF/UiO-66-NH_2_ for Chemical Protection and Decontamination. ACS Appl. Mater. Interfaces.

[34] Wu Y., Li F., Liu H., Zhu W., Teng M., Jiang Y., Li W., Xu D., He D., Hannam P. (2012). Electrospun fibrous mats as skeletons to produce free-standing MOF membranes. J. Mater. Chem..

[35] Zhang Y., Zhang Y., Wang X., Yu J., Ding B. (2018). Ultrahigh Metal-Organic Framework Loading and Flexible Nanofibrous Membranes for Efficient CO_2_ Capture with Long-Term, Ultrastable Recyclability. ACS Appl. Mater. Interfaces.

[36] Zhang Y., Yuan S., Feng X., Li H., Zhou J., Wang B. (2016). Preparation of Nanofibrous Metal-Organic Framework Filters for Efficient Air Pollution Control. J. Am. Chem. Soc..

[37] Wang C., Wang H., Luo R., Liu C., Li J., Sun X., Shen J., Han W., Wang L. (2017). Metal-organic framework one-dimensional fibers as efficient catalysts for activating peroxymonosulfate. Chem. Eng. J..

[38] Kim H., Kim M., Lee W., Kim S. (2018). Rapid removal of radioactive cesium by polyacrylonitrile nanofibers containing Prussian blue. J. Hazard. Mater..

[39] Xi J., Xia Y., Xu Y., Xiao J., Wang S. (2015). (Fe, Co)@nitrogen-doped graphitic carbon nanocubes derived from polydopamine-encapsulated metal-organic frameworks as a highly stable and selective non-precious oxygen reduction electrocatalyst. Chem. Commun..

[40] Zhang M., Wang C., Luo R., Liu C., Li J., Sun X., Shen J., Han W., Wang L. (2018). Metal–organic framework derived Co_3_O_4_/C@SiO_2_ yolk–shell nanoreactors with enhanced catalytic performance. J. Mater. Chem. A.

[41] Luo R., Liu C., Li J., Wang J., Hu X., Sun X., Shen J., Han W., Wang L. (2017). Nanostructured CoP: An efficient catalyst for degradation of organic pollutants by activating peroxymonosulfate. J. Hazard. Mater..

[42] Anipsitakis G.P., Dionysiou D.D. (2004). Transition metal/UV-based advanced oxidation technologies for water decontamination. Appl. Catal. B Environ..

[43] Xu Y., Ai J., Zhang H. (2016). The mechanism of degradation of bisphenol A using the magnetically separable CuFe_2_O_4_/peroxymonosulfate heterogeneous oxidation process. J. Hazard. Mater..

[44] Sharma J., Mishra I.M., Dionysiou D.D., Kumar V. (2015). Oxidative removal of Bisphenol A by UV-C/peroxymonosulfate (PMS): Kinetics, influence of co-existing chemicals and degradation pathway. Chem. Eng. J..

[45] Du Y., Ma W., Liu P., Zou B., Ma J. (2016). Magnetic CoFe_2_O_4_ nanoparticles supported on titanate nanotubes (CoFe_2_O_4_/TNTs) as a novel heterogeneous catalyst for peroxymonosulfate activation and degradation of organic pollutants. J. Hazard. Mater..

[46] Deng L., Shi Z., Zou Z., Zhou S. (2017). Magnetic EDTA functionalized CoFe_2_O_4_ nanoparticles (EDTA- CoFe_2_O_4_) as a novel catalyst for peroxymonosulfate activation and degradation of Orange G. Environ. Sci. Pollut. Res. Int..

[47] Zhu Z., Ji C., Zhong L., Liu S., Cui F., Sun H., Wang W. (2017). Magnetic Fe–Co crystal doped hierarchical porous carbon fibers for removal of organic pollutants. J. Mater. Chem. A.

[48] Lin K.Y., Chang H.A., Chen R.C. (2015). MOF-derived magnetic carbonaceous nanocomposite as a heterogeneous catalyst to activate oxone for decolorization of Rhodamine B in water. Chemosphere.

[49] Ji Y., Dong C., Kong D., Lu J. (2015). New insights into atrazine degradation by cobalt catalyzed peroxymonosulfate oxidation: Kinetics, reaction products and transformation mechanisms. J. Hazard. Mater..

[50] Huang G.X., Wang C.Y., Yang C.W., Guo P.C., Yu H.Q. (2017). Degradation of Bisphenol A by Peroxymonosulfate Catalytically Activated with Mn_1.8_Fe_1.2_O_4_ Nanospheres: Synergism between Mn and Fe. Environ. Sci. Technol..

[51] Huang Z., Bao H., Yao Y., Lu W., Chen W. (2014). Novel green activation processes and mechanism of peroxymonosulfate based on supported cobalt phthalocyanine catalyst. Appl. Catal. B Environ..

[52] Gong C., Chen F., Yang Q., Luo K., Yao F., Wang S., Wang X., Wu J., Li X., Wang D. (2017). Heterogeneous activation of peroxymonosulfate by Fe-Co layered doubled hydroxide for efficient catalytic degradation of Rhoadmine B. Chem. Eng. J..

[53] Hu L., Zhang G., Liu M., Wang Q., Wang P. (2018). Optimization of the catalytic activity of a ZnCo_2_O_4_ catalyst in peroxymonosulfate activation for bisphenol A removal using response surface methodology. Chemosphere.

[54] Wang C., Kim J., Malgras V., Na J., Lin J., You J., Zhang M., Li J., Yamauchi Y. (2019). Metal-organic frameworks and their derived materials: Emerging catalysts for sulfate radicals-based advanced oxidation process in water purification. Small.

