# Supplementary material for: Spiderweb-Like Fe-Co Prussian Blue Analogue Nanofibers as Efficient Catalyst for Bisphenol-A Degradation by Activating Peroxymonosulfate
Source: Nanomaterials (Basel). 2019 Mar 10;9(3):402. doi: 10.3390/nano9030402 (PMC6473942; doi:10.3390/nano9030402)
Supplement: Supplementary file 1 [file nanomaterials-09-00402-s001.pdf]

Supplementary Information

Spiderweb-Like Fe-Co Prussian Blue Analogue Nanofibers as Efficient Catalyst for Bisphenol-A Degradation by Activating Peroxymonosulfate

Hongyu Wang [1,2], Chaohai Wang [1,2], Junwen Qi [1,2,*], Yubo Yan [3], Ming Zhang [1,2], Xin Yan [1,2], Xiuyun Sun [1,2], Lianjun Wang [1,2] and Jiansheng Li [1,2,*]

[1] Key Laboratory of New Membrane Materials, Ministry of Industry and Information Technology, Nanjing University of Science & Technology, Nanjing 210094, China; wanghy4113@163.com (H.W.); wch2016@njust.edu.cn (C.W.); mzhang925@njust.edu.cn (M.Z.); 18205150297@163.com (X.Y.); sunxyun@njust.edu.cn (X.S.); wanglj@njust.edu.cn (L.W.)

[2] Jiangsu Key Laboratory of Chemical Pollution Control and Resources Reuse, School of Environmental and Biological Engineering, Nanjing University of Science & Technology, Nanjing 210094, China

[3] Jiangsu Engineering Laboratory for Environment Functional Materials, Huaiyin Normal University, Huaian 223300, China; yubo.yan@outlook.com

* Correspondence: qijunwen@njust.edu.cn (J.Q.); lijsh@njust.edu.cn (J.L.); Tel.: (+86)-025-8431-5351 (J.L.)

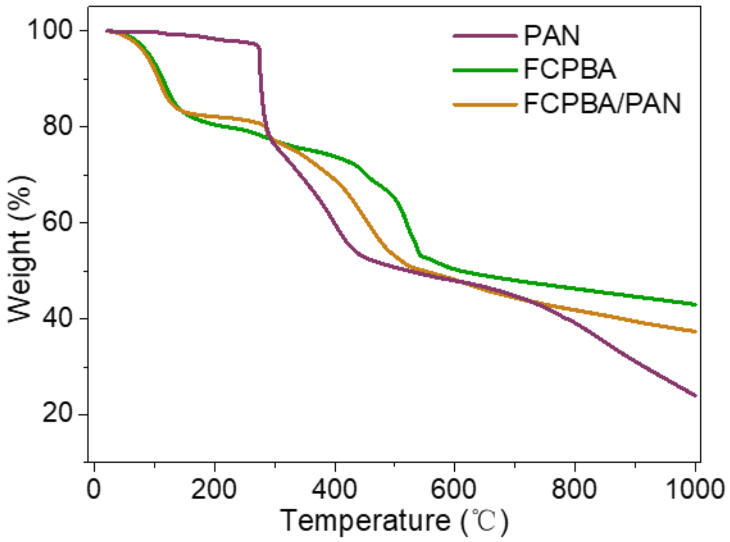

Figure S1. (Thermogravimetric analysis) TG curves of PAN, FCPBA, FCPBA/PAN.

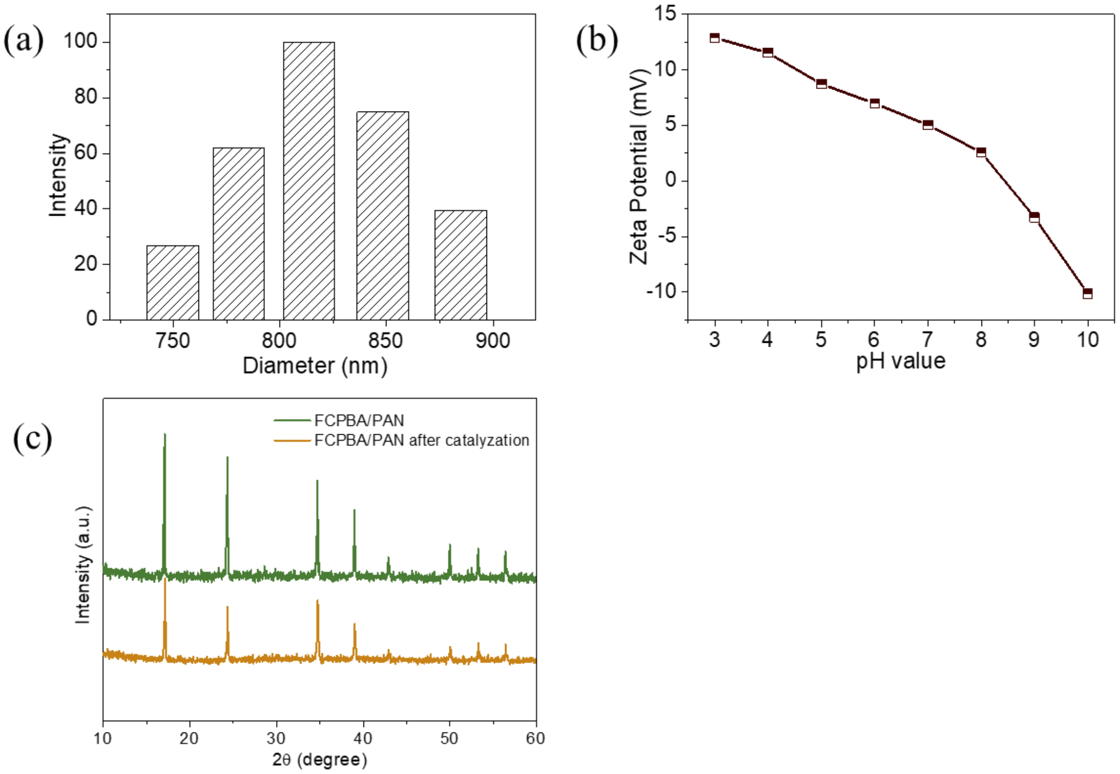

Figure S2. (a) Particle size; **(b)** zeta potential and **(c)** stability of FCPBA.

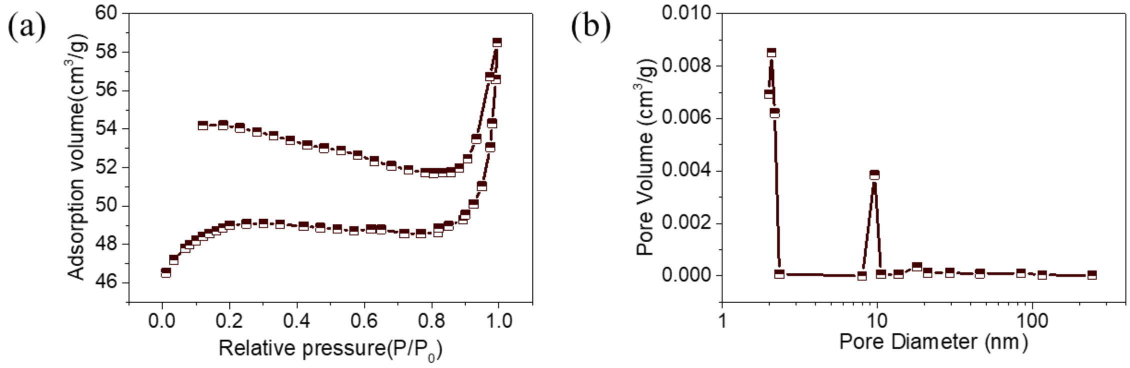

Figure S3. (a) N_2 sorption isotherms and **(b)** pore size distribution of FCPNA/PAN nanofibers.

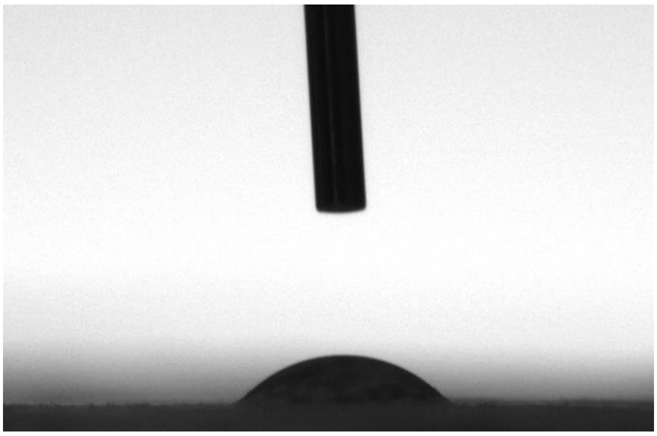

Figure S4. Water contact angle of FCPBA/PAN nanofibers.

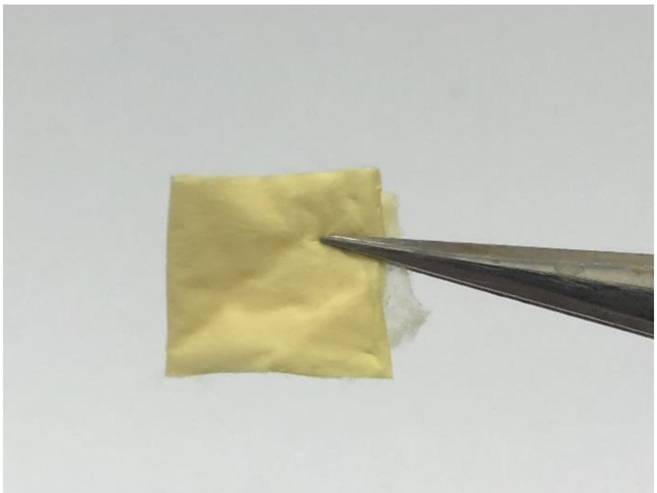

Figure S5. Mechanical properties of FCPBA/PAN nanofibers.

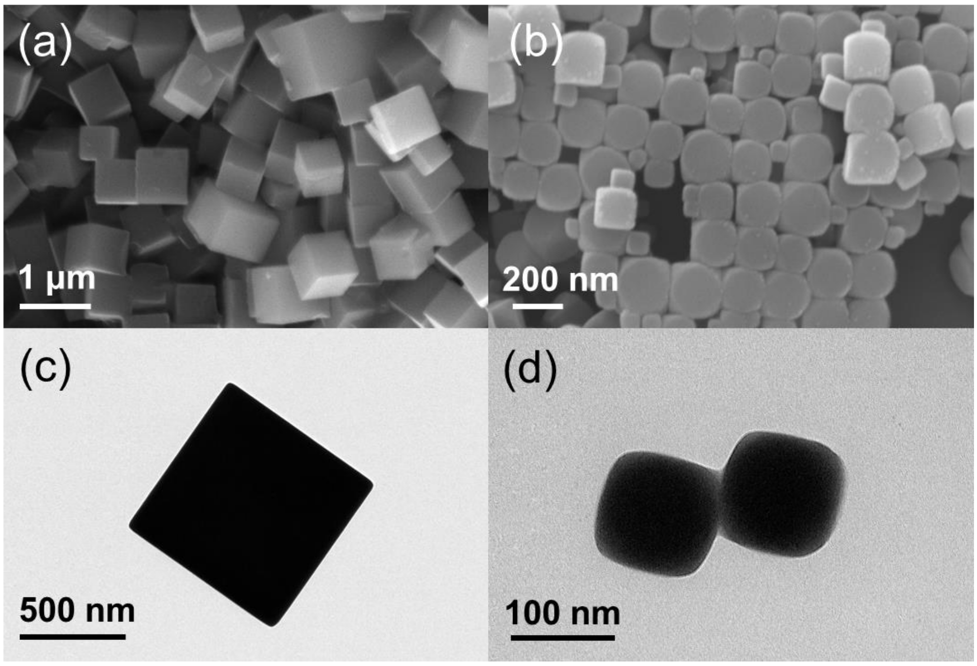

Figure S6. SEM images of (a) Fe-Fe PBA and (b) Co-Co PBA; TEM imagers of (c) Fe-Fe PBA and (d) Co-Co PBA.

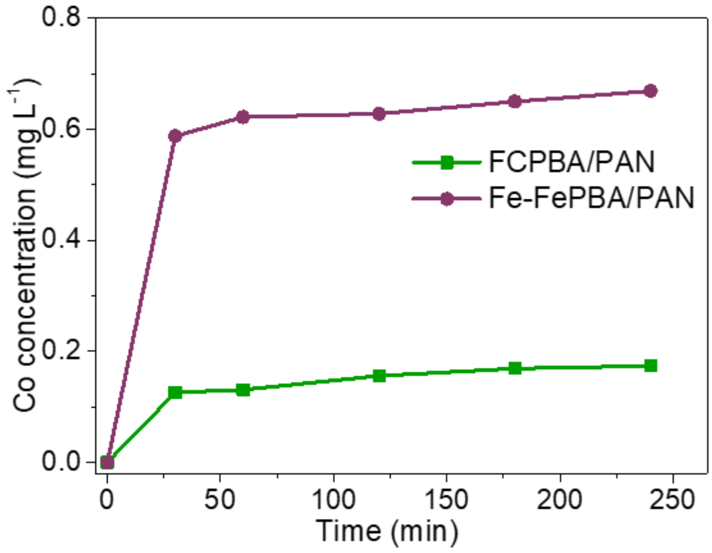

Figure S7. Cobalt leaching of FCPBA/PAN and Fe-FePBA/PAN in reaction system.
